# The use of electrical impedance tomography for individualized ventilation strategy in COVID-19: a case report

**DOI:** 10.1186/s12890-021-01411-y

**Published:** 2021-01-22

**Authors:** Zhanqi Zhao, Jin-Shou Zhang, Ying-Tzu Chen, Hou-Tai Chang, Yeong-Long Hsu, Inéz Frerichs, Andy Adler

**Affiliations:** 1 Department of Biomedical Engineering, Fourth Military Medical University, Xi’an, China; 2 Institute of Technical Medicine, Furtwangen University, Villingen-Schwenningen, Germany; 3 Division of Chest Medicine, Far Eastern Memorial Hospital, Taipei, Taiwan; 4 Department of Critical Care Medicine, Far Eastern Memorial Hospital, No. 21, Sec. 2, Nanya S. Rd., Banciao Dist., New Taipei City, 220 Taiwan, ROC; 5 Department of Anesthesiology and Intensive Care Medicine, University Medical Center of Schleswig-Holstein Campus Kiel, Kiel, Germany; 6 Department of Systems and Computer Engineering, Carleton University, Ottawa, Canada

**Keywords:** COVID-19, Electrical impedance tomography, Acute respiratory distress syndrome, Titrate of positive end-expiratory pressure, Personalizing ventilation strategies

## Abstract

**Background:**

Clinical management of COVID-19 requires close monitoring of lung function. While computed tomography (CT) offers ideal way to identify the phenotypes, it cannot monitor the patient response to therapeutic interventions. We present a case of ventilation management for a COVID-19 patient where electrical impedance tomography (EIT) was used to personalize care.

**Case presentation:**

The patient developed acute respiratory distress syndrome, required invasive mechanical ventilation, and was subsequently weaned. EIT was used multiple times: to titrate the positive end-expiratory pressure, understand the influence of body position, and guide the support levels during weaning and after extubation. We show how EIT provides bedside monitoring of the patient´s response to various therapeutic interventions and helps guide treatments.

**Conclusion:**

EIT provides unique information that may help the ventilation management in the pandemic of COVID-19.

## Background

COVID-19 can lead to respiratory failure where patients might have different phenotypes that need various ventilation strategies [1]. While computed tomography (CT) offers ideal way to identify the phenotypes, it cannot monitor the patient response to therapeutic interventions. Electrical impedance tomography (EIT) is a novel bedside tool that can monitor ventilation distribution and guide individualized ventilation strategies [2]. A recent study suggested that EIT can assess the lung recruitability in COVID-19 patients [3]. In the present case, we would like to report the use of EIT for personalizing ventilation strategies at multiple stages during the treatment of COVID-19 associated acute respiratory distress syndrome (ARDS). EIT was used during invasive mechanical ventilation, weaning from ventilator and oxygen therapy after extubation.

## Case presentation

### Patient history

A 53-year-old male was sent to Far Eastern Memorial Hospital, Taiwan, due to shortness of breath for ~ 2 weeks and pneumonia confirmed by chest X-ray. Disease history included sleep apnoea, coronary artery disease after percutaneous occlusion balloon angioplasty and medication controlled hypertension. SARS-CoV-2 was confirmed positive. With episodes of dyspnoea, progressive tachypnoea, paradoxical respiratory pattern and impaired consciousness, the patient was transferred to the ICU and intubated on March 28, 2020. EIT measurements were performed throughout the disease management to facilitate decision making. During invasive ventilation, EIT was used to titrate positive end-expiratory pressure (PEEP) and body positioning. EIT was next used to manage the weaning support level, and finally to determine an appropriate flow rate for high flow nasal cannula (HFNC) oxygen therapy.

### PEEP titration

The patient was ventilated under volume controlled mode with the following initial settings: tidal volume 400 ml, respiratory rate 20 /min, FiO_2_ 1.0, PEEP 12 cmH_2_O. The peak and plateau pressures were 43 and 23 cmH_2_O, respectively. PaO_2_ was 229.0 mmHg and the measured respiratory system mechanics were: resistance 24.5 cmH_2_O/L/s, compliance 38.4 mL/cmH_2_O. Intrinsic PEEP of 0.7 cmH_2_O was observed. PEEP was adjusted to 10 cmH_2_O at a later time point. Chest X-ray showed infiltrates progressed in both lungs, and pleural effusion especially in the left lung (Additional file 1: Fig. S1 of the online supplement). Co-infection with other viruses was excluded. Medications and body temperature are summarized in the online supplement (Additional file 1: Fig. S2).

**Fig. 1:**
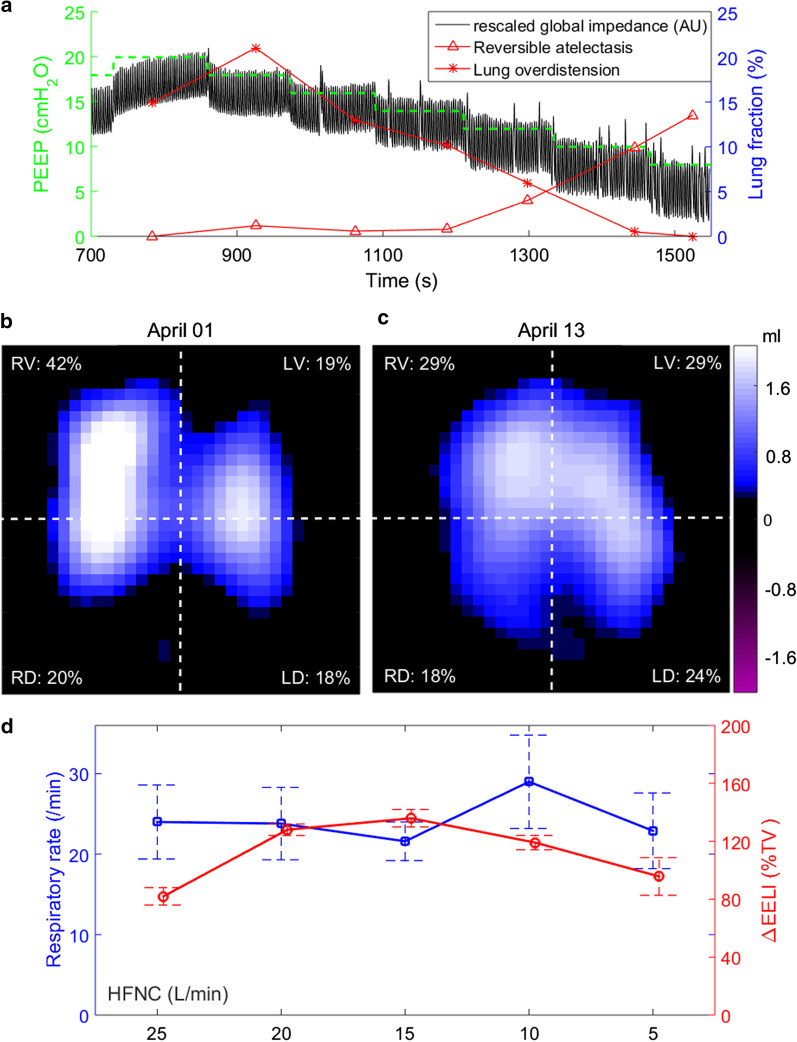
Use of EIT during invasive mechanical ventilation, weaning from ventilator and oxygen therapy after extubation. **a** Titration of positive end-expiratory pressure (PEEP) with cumulated regional collapse and overdistension based on EIT. **b** Functional EIT (fEIT) image illustrated the tidal ventilation distribution on April 1st indicating poor ventilation in the dorsal regions and left lung. RV, right ventral lung regions; LV, left ventral lung regions; RD, right dorsal lung regions; LV, left dorsal lung regions. Highly ventilated regions are marked in light blue and white. EIT measurements were performed in supine position. **c** fEIT on April 13th before spontaneous breathing trial showing more homogenous distribution of ventilation. **d** Change of respiratory rate and end-expiratory lung impedance (EELI) during high flow nasal cannula (HFNC) with various flows. ΔEELI was calculated relative to the EELI during a short period with zero support and proportional to the tidal variation during the same period (%TV). Medians and interquartile ranges of respiratory rate and ΔEELI values during the last minute of each phase are plotted

The protocol for EIT-guided PEEP titration increases pressure starting at 8 cmH_2_O with 2-min steps of 2 cmH_2_O till 20 cmH_2_O, and then decreases it with 2 cmH_2_O steps [4]. At each step, fractions of regional collapse and overdistention are calculated and the PEEP level selected (12 cmH_2_O) at the intercept point, to provide the best compromise between collapsed and overdistended lung (Fig. 1a). PEEP was tapered gradually following this procedure of [4].

### Positioning

Continuous lateral rotation therapy was provided to the patient [5]. Lateral (both left and right, ~ 40° with pillows) and supine positions were performed (~ 2 h for each position). A routine blood gas analysis after the left lateral position on April 1st indicated worsening of gas exchange. EIT revealed that ventilation in the left lung was much worse than the right lung (Fig. 1b). Twelve hours after stopping left lateral positioning (only right lateral and supine), PaO_2_ raised from 90 to 130 mmHg.

### Weaning support level

As the patient’s condition gradually stabilized, improvement of ventilation was observed (Fig. 1c). Nasal swab test for SARS-CoV-2 showed negative, the patient started to wean from ventilator on April 13. Automatic tubing compensation (ATC) was used for a spontaneous breathing trial (SBT). EIT was used to select a suitable support level with a previously described method [6]. Three support levels were tested, namely ATC100%, ATC70% and ATC0%. The patient was first ventilated under assisted-control mode with PEEP of 8 cmH_2_O. SBT was started with ATC100% for 5 min, followed by ATC70% and ATC0% with PEEP kept at 8 cmH_2_O. The increase of intra tidal ventilation distribution towards dorsal regions was noted as the support level decreased from AC to ATC100% further to ATC70%. However, no further increase was observed when the level changed from ATC70% to ATC0%, indicated a possible fatigue of diaphragm [6] (See Additional file 1: Fig. S3). ATC70% was considered as an adequate support level without overloading the work for respiratory muscles. The patient was successfully extubated after 18 days of invasive mechanical ventilation on April 15th.

### High flow nasal cannula (HFNC)

After extubation, HFNC was provided and EIT used to select an appropriate flow rate (Precision flow, Vapotherm, NH, US. Figure 1d) and to test whether nasal cannula could be used instead of HFNC. The flow rate decreased from 25 to 5 L/min with steps of 5 L/min and 10 min of each step. Respiratory rate was the lowest and end-expiratory lung impedance (corresponding to lung volume) was the highest at 15 L/min, at which the patient reported being most comfortable. As the patient’s condition improved, he was transferred to general ward on April 18th.

## Discussion and conclusions

In the present case, we highlighted the use of EIT throughout the clinical management of ventilation in a ARDS patient with COVID-19. Personalized EIT-based strategies were successfully developed for PEEP titration, positioning, support levels of weaning and HFNC after extubation.

Infection control is a critical issue of COVID-19, and 3.8% of healthcare workers in China, were infected [7]. Keeping monitoring equipment at the bedside to avoid infection during transportation of patients is important, and EIT may fill the gap. In a recent guideline on the management of COVID-19 patients, high PEEP and prone position were suggested [8]. However, high PEEP may induce overdistension and barotrauma, whereas prone position may not be beneficial for all subjects. EIT-guided PEEP titration is easy to implement, and might improve clinical outcomes compared to traditional pressure–volume loops [4]. We have used the intercept point to balance overdistension and collapse but other criteria, e.g. limiting collapse or overdistension percentages, may also be considered, depending on the treatment objective. The surviving sepsis campaign guidelines advised against stepwise recruitment maneuvers with the example of increasing PEEP from 25 to 45 cmH_2_O [8]. Although we used stepwise recruitment maneuver, such high PEEP is strictly forbidden in our internal protocol. Interestingly, the role of EIT during PEEP titration has been proposed by using interspersed short diagnostic recruitment maneuvers in order to infer on the potential of lung recruitment [9, 10]. Previous EIT studies have demonstrated the potential of guiding individual positioning in various patients (e.g. [11]). In the present case, the worsening of blood gasses was puzzling, since the ventilation defect in the left lung compared to right lung was not expected based on the chest X-ray (Additional file 1: Fig. S4). Functional EIT images clearly showed the ventilation distribution, which facilitated the decision making of avoiding left lateral positioning.

EIT helps to predict weaning outcome by assessing the diaphragm recovery [6]. Based on the rationale, we used EIT to select a suitable support level for SBT, which contributed to the successful weaning after prolonged mechanical ventilation for the present case. For ventilation support after extubation, it is important to monitor the respiratory rate and potential changes of lung volume, which could be estimated by the changes of lung impedance measured with EIT. A recent study indicated that overdistension may also occur during HFNC [12]. Combining the detection of pendelluft [13], bedside EIT may provide further insight on lung status, which might be helpful to prevent patient self-inflicted lung injury. Besides the application of selecting a flow rate for HFNC, EIT could also be used to monitor the lung recovery and to identify the need of reintubation.

The pandemic of COVID-19 is still a current concern at a global level. The daily use of EIT may provide unique information to direct the ventilation management throughout patient ICU stay, from the time of intubation during mechanical ventilation until the time of weaning, to the management of oxygen therapy after extubation in COVID-19 patients. More importantly, this information is provided at the bedside to enable monitoring and adjustment of ventilation management in a timely manner.

## Supplementary Information

**Additional file 1.** EIT data analysis and additional patient information.

## Data Availability

The data are with the authors and will be available upon reasonable request.

## Abbreviations

ATC: Automatic tube compensation
ARDS: Acute respiratory distress syndrome
CT: Computed tomography
EIT: Electrical impedance tomography
FiO_2_: Fractional inspired oxygen
HFNC: High-flow nasal cannula
ICU: Intensive care unit
ITVD: Intra tidal ventilation distribution
PaO_2_: Arterial oxygen partial pressure
PEEP: Positive end-expiratory pressure

## References

[1] Gattinoni L, Chiumello D, Rossi S (2020). COVID-19 pneumonia: ARDS or not?. Crit Care.

[2] Zhao Z, Fu F, Frerichs I (2020). Thoracic electrical impedance tomography in Chinese hospitals: a review of clinical research and daily applications. Physiol Meas.

[3] Mauri T, Spinelli E, Scotti E, Colussi G, Basile MC, Crotti S, Tubiolo D, Tagliabue P, Zanella A, Grasselli G (2020). Potential for lung recruitment and ventilation-perfusion mismatch in patients with the acute respiratory distress syndrome from coronavirus disease 2019. Crit Care Med.

[4] Zhao Z, Chang MY, Gow CH, Zhang JH, Hsu YL, Frerichs I, Chang HT, Moller K (2019). Positive end-expiratory pressure titration with electrical impedance tomography and pressure-volume curve in severe acute respiratory distress syndrome. Ann Intensive Care.

[5] Staudinger T, Bojic A, Holzinger U, Meyer B, Rohwer M, Mallner F, Schellongowski P, Robak O, Laczika K, Frass M (2010). Continuous lateral rotation therapy to prevent ventilator-associated pneumonia. Crit Care Med.

[6] Zhao Z, Peng SY, Chang MY, Hsu YL, Frerichs I, Chang HT, Moller K (2017). Spontaneous breathing trials after prolonged mechanical ventilation monitored by electrical impedance tomography: an observational study. Acta Anaesthesiol Scand.

[7] Wu Z, McGoogan JM (2020). Characteristics of and important lessons from the coronavirus disease 2019 (COVID-19) Outbreak in China: summary of a report of 72314 cases from the chinese center for disease control and prevention. JAMA.

[8] Alhazzani W, Moller MH, Arabi YM, Loeb M, Gong MN, Fan E, Oczkowski S, Levy MM, Derde L, Dzierba A (2020). Surviving sepsis campaign: guidelines on the management of critically ill adults with coronavirus disease 2019 (COVID-19). Crit Care Med.

[9] Becher T, Buchholz V, Schadler D, Frerichs I, Weiler N (2016). Electrical impedance tomography-based algorithm for personalized adjustment of mechanical ventilation in ARDS patients. Intensive Care Med Exp.

[10] Rezoagli E, Bellani G (2019). How I set up positive end-expiratory pressure: evidence- and physiology-based!. Crit Care.

[11] Lehmann S, Leonhardt S, Ngo C, Bergmann L, Schrading S, Heimann K, Wagner N, Tenbrock K (2018). Electrical impedance tomography as possible guidance for individual positioning of patients with multiple lung injury. Clin Respir J.

[12] Zhang R, He H, Yun L, Zhou X, Wang X, Chi Y, Yuan S, Zhao Z (2020). Effect of postextubation high-flow nasal cannula therapy on lung recruitment and overdistension in high-risk patient. Crit Care.

[13] Sang L, Zhao Z, Yun PJ, Frerichs I, Möller K, Fu F, Liu X, Zhong N, Li Y (2020). Qualitative and quantitative assessment of pendelluft: a simple method based on electrical impedance tomography. Ann Transl Med.

