# Supplementary material for: The use of electrical impedance tomography for individualized ventilation strategy in COVID-19: a case report
Source: BMC Pulm Med. 2021 Jan 22;21:38. doi: 10.1186/s12890-021-01411-y (PMC7820832; doi:10.1186/s12890-021-01411-y)
Supplement: Supplementary file 1 — Additional file 1. EIT data analysis and additional patient information. [file 12890_2021_1411_MOESM1_ESM.pdf]

Online Supplement

The use of electrical impedance tomography for individualized ventilation strategy in COVID-19: a case report

Zhanqi Zhao, Jin-Shou Zhang, Ying Tzu Chen, Hou-Tai Chang, Yeong-Long Hsu, Inéz Frerichs, Andy Adler

EIT data analysis

PEEP titration:

Regional compliance was computed in all pixels in the lung regions at each PEEP level as ΔZ/ΔP, where ΔZ is the tidal impedance variation and ΔP is the driving pressure. Then, cumulated collapse and overdistension percentages were estimated based on the decrease of regional compliance curve during decremental PEEP titration, either toward lower or higher PEEP levels [1].

Assuming $C_{reg\_i\_MAX} = \max(\Delta Z_{i\_allPEEP} / \Delta P_{aw})$

$$Col_i = \frac{C_{reg\_i\_MAX} - \Delta Z_{i\_currentPEEP} / \Delta P_{aw}}{C_{reg\_i\_MAX}} \times 100\% \qquad (1)$$

If current PEEP is lower than the PEEP level where $C_{reg\_i\_MAX}$ is located, then Col_i represents the collapsed percentage ($i \in lung$). If current PEEP is higher than the level of $C_{reg\_i\_MAX}$, Col_i can be substituted by another term $Over_i$ and represents the overdistension percentage. The cumulated collapsed or overdistension can be calculated as follows:

$$Col_{cum} = \frac{\sum (Col_i \times C_{reg\_i\_MAX})}{\sum C_{reg\_i\_MAX}} \qquad (2)$$

Similarly, Col_{cum} represents collapsed or be substituted by $Over_{cum}$ and $Over_i$ representing overdistension, depending on the current PEEP and the PEEP where $C_{reg\_i\_MAX}$ is located.

The PEEP level selected for the patients in the EIT group was the intercept point of cumulated collapse and overdistension percentages curves, providing the best compromise between collapsed and overdistended lung.

Weaning support level determined with intra tidal ventilation distribution (ITVD)

Let Z_n be the EIT frames dividing inspiration into eight iso-volume slices (n=1, 2, …, 7). Z_0 and Z_8 represent the start and end of inspiration, respectively. Instead of calculating the tidal image, iso-volume images ΔZ_n are built by subtracting Z_{n-1} from Z_n (n=1, 2,…, 8). The ITVD is calculated for each ROI and plotted against each other.

$$ITVD_{ROI} = \frac{\Delta Z_{n\_ROI}}{\Delta Z_n} \quad (3)$$

Where ΔZ_{n_ROI} is the sum of impedance changes in the ROIs at n, ΔZ_n is the sum of all ROIs (n=1, 2,…, 8). A variation of this measure was proposed to divide inspiration with equal time length instead of iso-volume and to calculate the differential images $\Delta Z_n'$ by subtracting Z_0 from Z_n (n=1, 2,…, 8).

Previous studies showed that spontaneous effort increases the distribution of the dorsal regions, which could be used to assess the diaphragm function [2, 3]. In our present case, three support levels were tested, namely ATC100%, ATC70% and ATC0%. The patient was first ventilated under assisted-control mode with PEEP of 8 cmH_2O. SBT was started with ATC100% for 5 minutes, followed by ATC70% and ATC0% with PEEP kept at 8 cmH_2O. the increase of intra tidal ventilation distribution towards dorsal regions was noted as the support level decreased from AC to ATC100% further to ATC70% (Fig. S3). However, no further increase was observed when the level changed from ATC70% to ATC0%, indicated a possible fatigue of diaphragm.

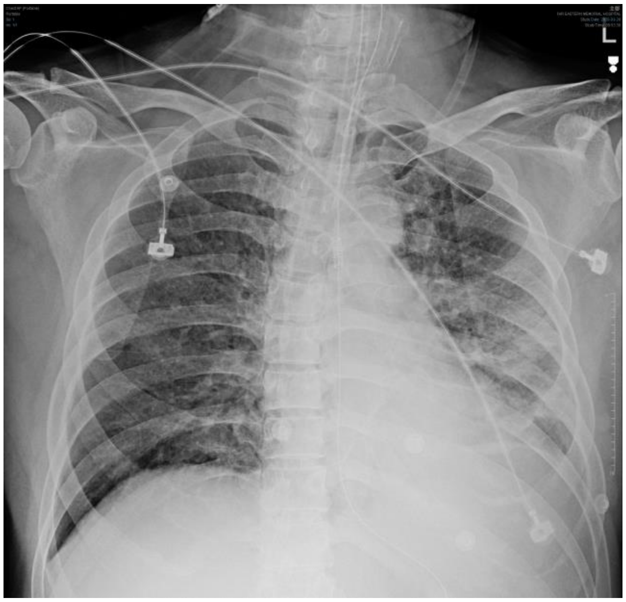

Figure S1. Chest x-ray on the day of ICU admission.

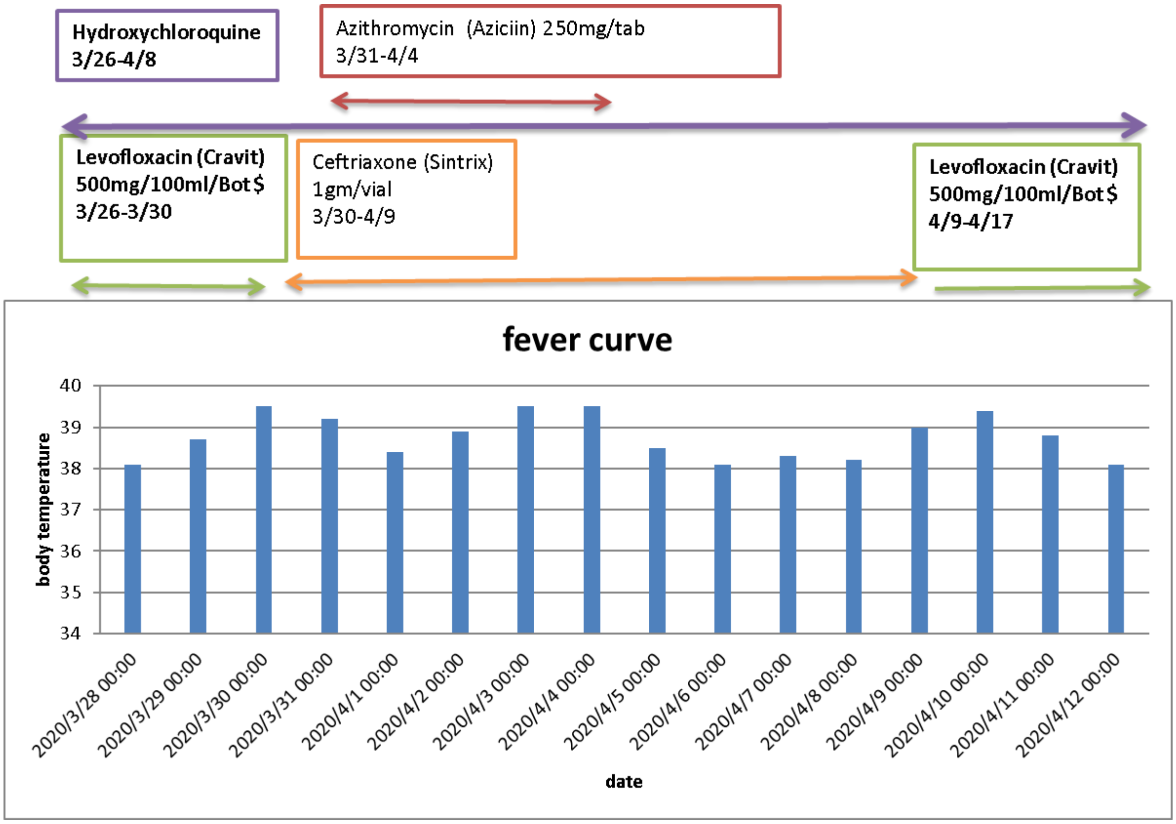

Figure S2. Medications and the change of body temperature from the day of intubation till the clearance of SARS-CoV-2.

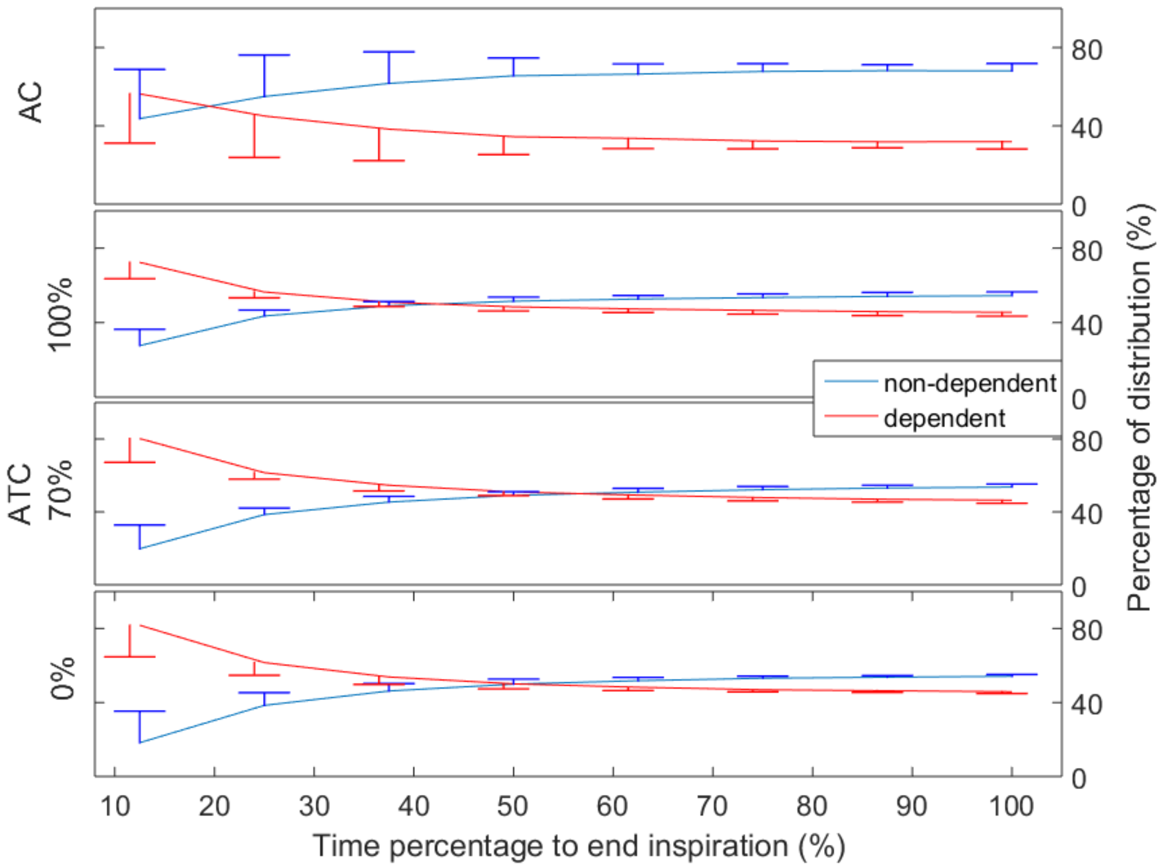

Figure S3. Intra tidal ventilation distribution (ITVD) between dependent and non-dependent regions during assist-control (AC) and spontaneous breathing trial with various support levels (ATC100% - 0%). Percentages of ventilation distributed in non-dependent (ventral, blue lines) and dependent (dorsal, red lines) regions were calculated at eight time points during inspiration (X axis, 0%, beginning of inspiration; 100%, end of inspiration). Medians and interquartile ranges of ITGD values during the last minute of each phase are plotted.

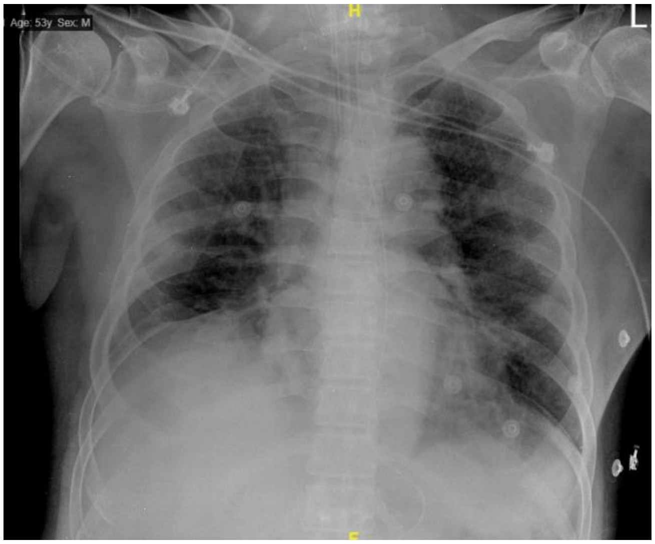

Figure S4. Chest x-ray on April 1st

References

1. Costa EL, Borges JB, Melo A, Suarez-Sipmann F, Toufen C, Jr., Bohm SH, et al. Bedside estimation of recruitable alveolar collapse and hyperdistension by electrical impedance tomography. Intensive Care Med. 2009; **35**(6): 1132-7.

2. Zhao Z, Peng SY, Chang MY, Hsu YL, Frerichs I, Chang HT, et al. Spontaneous breathing trials after prolonged mechanical ventilation monitored by electrical impedance tomography: an observational study. Acta Anaesthesiol Scand. 2017; **61**(9): 1166-75.

3. Frerichs I, Amato MB, van Kaam AH, Tingay DG, Zhao Z, Grychtol B, et al. Chest electrical impedance tomography examination, data analysis, terminology, clinical use and recommendations: consensus statement of the TRanslational EIT developmeNt stuDy group. Thorax. 2017; **72**(1): 83-93.
